# Surface Modification of Basalt Fibres with ZnO Nanorods and Its Effect on Thermal and Mechanical Properties of PLA-Based Composites

**DOI:** 10.3390/biom11020200

**Published:** 2021-02-01

**Authors:** Francesca Sbardella, Andrea Martinelli, Valerio Di Lisio, Irene Bavasso, Pietro Russo, Jacopo Tirillò, Fabrizio Sarasini

**Affiliations:** 1 Department of Chemical Engineering Materials Environment, Sapienza-Università di Roma & UdR INSTM, Via Eudossiana 18, 00184 Roma, Italy; irene.bavasso@uniroma1.it (I.B.); jacopo.tirillo@uniroma1.it (J.T.); 2 Department of Chemistry, Sapienza-Università di Roma, P.le A. Moro, 5, 00185 Roma, Italy; valerio.dilisio@uniroma1.it; 3 Institute for Polymers, Composites, and Biomaterials, National Council of Research, Via Campi Flegrei 34, 80078 Pozzuoli, Italy; pietro.russo@unina.it

**Keywords:** basalt fibre, poly(lactic acid), interfacial adhesion, crystallization, ZnO nanorods, biocomposites

## Abstract

The composites based on basalt fibres and poly(lactic acid) (PLA) show promising applications in biomedical and automotive fields, but their mechanical performance is still largely hindered by poor interfacial properties. Zinc oxide nanorods have been successfully used to tune the PLA/basalt fibre interface by growing them on commercially available basalt fabrics. The hierarchical fibres significantly enhanced the mechanical properties of PLA-based composites, especially their flexural strength and stiffness. These values are 26% and 22% higher than those of unmodified basalt/PLA composites, and 24% and 34% higher than those of glass/PLA composites used as a baseline. The increase in tensile and flexural properties hinges on the mechanical interlocking action promoted by ZnO nanorods and on the creation of a compact transcrystallinity structure. A degradation of PLA matrix was detected but it was positively counteracted by the better interfacial stress transfer. This study offers a novel approach for modifying the fibre–matrix interface of biocomposites intended for high-performance applications.

## 1. Introduction

In recent years, there is an increasing urgency to develop new greener products and innovative technologies that can reduce the dependence on fossil fuel. In the fibre-reinforced composite materials industry, this is possible by combining fibres of natural origin with polymers derived from crops (bioplastics), offering a solution to the imminent eco-environmental threat [1]. The use of bioplastics, intended as polymers derived from renewable sources, is gradually included in the world of composite materials as an excellent alternative to materials based on nonrenewable sources. Poly(lactic acid) (PLA) is a thermoplastic polymer produced by ring-opening polymerization of lactide derived from the fermentation of starch and sugar. It is the most popular candidate in the bioplastic family thanks to the numerous advantages such as biocompatibility, biodegradability and ease of processing [2,3]. In the field of materials for semistructural applications, the mechanical and thermomechanical properties of a polymer are fundamental, which are generally highly dependent on crystallinity and molecular weight. Unfortunately, one of the most significant disadvantages of PLA is its poor ability to form a crystalline structure and the consequent relatively low thermomechanical stability (glass transition temperature *T*_g_ ~ 60 °C) [4]. To obtain a durable structural material it is necessary to reduce these disadvantages that limit PLA applications in many areas. In this regard, the reinforcement of PLA with fibres is a topic widely investigated [5,6,7]. The fibres generally used as reinforcement in composite materials can be divided into two major classes: synthetic fibres (glass and carbon) and natural fibres, which can be of vegetable origin (flax, jute, cotton, and hemp) or of mineral origin (basalt). Various studies are reported in literature on the use of both synthetic [8,9,10] and natural fibres [11,12,13] as reinforcement in composites based on PLA.

Considering the current need to preserve the environment, the use of natural fibres that are biodegradable, recyclable, and lightweight is certainly preferable to synthetic fibres. Basalt fibre (BF), classified as a natural fibre, is obtained by melting the basaltic rock and has good chemical, corrosion resistance, and thermal stability and is a nontoxic and eco-friendly material. Its good mechanical properties and ability to be easily processed make it a valid alternative as reinforcing agent compared to the two main synthetic fibres, i.e., glass and carbon. It is known that basalt fibres have a greater tensile strength than E-glass fibres thus offering at least the same if not better performance, and, at the same time, drastically reducing costs compared to carbon fibres in addition to the fact that the deformation at break of basalt fibres is greater than that of carbon fibres [14,15].

Biodegradability together with satisfactory mechanical properties make BF/PLA composites as excellent materials in the field of medical equipment, bone repairing materials, automobile shell, etc. [15,16]. The success of composite materials is closely related to the excellent mechanical properties that are the result of a synergistic combination of their main constituents, and it depends greatly on the interfacial adhesion between the same. However, the compatibility between PLA matrix and BF is very poor, which leads to low interface performance and reduced mechanical properties for composites. Many studies have been conducted to improve the interfacial adhesion between basalt fibre and PLA matrix, focusing on various sizing or grafting methods, as well as chemical and plasma treatments [17,18,19,20]. Despite the good results, these treatments are often associated with a significant reduction in tensile strength of the underlying fibres. Indeed, Förster et al. have shown that the strength properties are significantly reduced, with a reduction on the average tensile strength of about 32–40%, from alkali and subsequent CVD (Chemical Vapour Deposition) treatments on basalt fibres [21]. For this reason, an alternative method to superficially modify the fibres is needed. The fibre whiskerization method, which involves the introduction of nanomaterials on the fibre surface, forming a hierarchical fibre, has various advantages such as the enhancement of the fibre surface area, the possibility of mechanical interlocking, wetting capillary by the matrix, and local reinforcement of the interphase [22]. There are many nanomaterials used to modify the surface of the fibres, including carbon nanotubes (CNT) [21], calcium carbonate (CaCO_3_) [23], silica (SiO_2_) [24], zinc oxide (ZnO) [25], as well as epoxy/silica nano-hybrid sizing [26]. Among these nanomaterials, particular attention has been recently placed on ZnO, due to its multiple properties, such as an extraordinary photocatalytic and antimicrobial activity and good chemical and thermal stability, making it one of the most used materials applicable in a wide range of industrial sectors [27]. Moreover, ZnO offers one of the largest varieties of particle structures among all known materials. It can occur in one-dimensional structures, which is the largest category, such as nanorods [28], nanoneedles [29], nanoribbons, nanotubes, nanowires [30], and in bi- and three-dimensional structures, nanoplates and flowers-like structures [31]. Because of its interesting properties, zinc oxide synthesis has been studied by numerous researchers developing a wide range of methodologies, including vapor deposition, precipitation in aqueous solution, sol–gel process, hydrothermal synthesis, and biological synthesis. [32,33,34,35]. Among these methods, the hydrothermal process does not need organic solvents and does not require high process temperatures, thus guaranteeing a relatively simple and environment-friendly methodology [36].

Recently, ZnO nanostructures demonstrated to be promising multifunctional whiskerization materials, when vertically synthesized on the surface of the reinforcing fibres, in particular carbon [37], glass [25], and basalt fibres [38]. ZnO nanofillers can be mixed with PLA to produce multifunctional biodegradable nanocomposites with important features such as antibacterial protection [39,40] or ultraviolet absorption [41,42]. Another important characteristic of zinc oxide is that it could be used as nucleating agent to increase the crystallinity of polymer. Several studies have been carried out on polymers such as polypropylene, nylon, and poly(ethylene terephthalate) (PET) [43]. Moreover, Wang et al. [44] have shown that even the fibres themselves play an important role in the crystallization of the polymer when are in contact with the matrix in a composite material. In fact, they tested both synthetic (carbon, PET, Kevlar, and glass) and natural fibres (hemp, flax, and cellulose) showing that the nucleating ability of different fibres toward PLA was successful. Recent investigations showed that the incorporation of basalt fibres into PLA matrix can be used to tailor the overall degree of crystallinity and heat deflection temperature [45] due to transcrystallization phenomena controlled by the stereoregularity of PLA [46].

In this work, attention is given to the eco-friendly aspect, producing an innovative hierarchical fibre composite material reinforced with basalt fibres in a PLA matrix. To improve the interfacial adhesion with PLA, the surface of basalt fabrics has been modified by creating a layer of ZnO nanorods, through a solvent-free and low temperature hydrothermal process. This intermediate layer is of substantial importance for producing more interlocking points with the matrix and, therefore, a better load transfer from the matrix to the fibre. The quasi-static mechanical properties of basalt/ZnO/PLA composites have been discussed and interpreted by considering the combined effects of basalt and ZnO nanorods on the crystallization of the resulting composites as obtained by differential scanning calorimetry (DSC).

## 2. Materials and Methods

### 2.1. Raw Materials

The substrate used is a commercial plain woven basalt fabric (220 g/m^2^) supplied by Basaltex, with a commercial sizing compatible with epoxy resin. Analytical grade reagents of zinc acetate dihydrate (Zn (CH_3_COO)_2_⋅2H_2_O, 99%), zinc nitrate hydrate (Zn (NO_3_)_2_⋅6H_2_O, 99%), sodium hydroxide (NaOH, 99%), and hexamethylenetetramine (HMTA, 99%) were obtained from Sigma-Aldrich and used without any further purification. The absolute ethanol (CH_3_CH_2_OH, 99.8%) was supplied by VWR Chemicals and the 18 MΩ·cm Milli-Q water was used throughout the experimental steps. A commercial extrusion-grade PLA (Ingeo^TM^ Biopolymers 4032D, NatureWorks) was used as matrix material (*ρ* = 1.24 g/cm^3^, MFR (210 °C, 2.16 kg) = 7 g/10 min). For comparison purposes, composites were also manufactured with a plain-woven glass fabric (200 g/m^2^) with a commercial sizing compatible with epoxy resin.

### 2.2. Synthesis of ZnO Nanorods on Basalt Fabrics

ZnO nanorods were synthesized following a two-step hydrothermal synthesis, with an initial formation of the seed layer and then the growth of ZnO nanorods through nucleation. The first step is the seeding part, where a solution A 1.25·10^−2^ M (1 mmol of Zn (O_2_CCH_3_)_2_(H_2_O)_2_ and 80 mL of EtOH) was prepared and heated at 50 °C with vigorous stirring for 5 min. Simultaneously, a solution B was prepared, by dissolving 1 mL of NaOH solution 0.2 M in 100 mL of EtOH (2·10^−3^ M), and heated for 5 min at 50 °C under vigorous stirring. After that reaction time, both solutions were cooled at room temperature. Then, 40 mL of solution A was added to 320 mL of EtOH, and 40 mL of solution B was added to 100 mL of EtOH (*V*_final_ = 500 mL). These solutions were heated separately until their temperature reached 65 °C, finally mixed to form the seeding solution and heated to 65 °C for 30 min with vigorous agitation. The basalt fabrics, which were previously washed with EtOH and dried at 150 °C in the oven for 10 min, were dipped in the seeding solution, maintaining the temperature at 65 °C, for 10 min. Finally, basalt fabrics were dried in an oven at 150 °C for 10 min. The second phase of this process consists of the growth of ZnO nanorods. A solution was prepared with 3.71 g of zinc nitrate hexahydrate and 1.75 g of hexamethylenetetramine, in 500 mL of Milli-Q water. This mixture was heated to 90 °C with vigorous stirring. Once the solution reached a temperature of 90 °C, the basalt fabrics, previously treated in the seeding solution, were immersed in the growth solution for 5 h. At the end of the growing step, the samples were rinsed with Milli-Q water to remove the presence of precipitates.

### 2.3. Morphological Characterization by Scanning Electron Microscopy (SEM)

The morphology of the ZnO-decorated basalt fabrics and of failed composite laminates was analysed by FE-SEM, a field-emission scanning electron microscope (MIRA3 by Tescan, Brno, Czech Republic). All specimens were sputter coated with chromium prior to each FE-SEM observation.

### 2.4. X-ray Diffraction (XRD) Analysis

The identification of the crystalline phases of ZnO was performed by X-ray diffraction (XRD) analysis by means of Philips X’Pert PRO powder diffractometer (Malvern Panalytical B.V., Almelo, Netherlands). XRD spectra were collected in the range of 2*θ* = 10°–80° with a step size of 0.02° and a time per step of 3 s. The employed radiation was monochromatic CuK_α_ (40 kV to 40 mA).

### 2.5. Static Contact Angle Measurements

The wetting properties of modified basalt fabrics were investigated by measuring the static contact angle with a video-based optical contact angle instrument, OCA 15 Pro, DataPhysics Instruments GmbH, Filderstadt, Germany). During this test, degassed water drops with a volume of 3 µL were applied to the basalt fabric. A minimum of 10 droplets localized on different areas of the basalt fabric samples were analysed. Contact angle values were determined by drop shape analysis using the DataPhysics SCA 20 software (DataPhysics Instruments GmbH, Filderstadt, Germany) module.

### 2.6. Composite Manufacturing

ZnO-decorated and neat basalt fabrics were used to manufacture composite laminates based on a PLA matrix by compression moulding (model P400E by Collin GmbH (Edersberg, Germany)). The PLA films (about 85 µm of thickness) were obtained using a Collin Teach-Line E20T extruder (COLLIN Lab & Pilot Solutions GmbH, Maitenbeth, Germany) equipped with a flat head die and connected to the Collin Teach-Line CR72T Flat film line (COLLIN Lab & Pilot Solutions GmbH, Maitenbeth, Germany). Before filming, PLA pellets were dried in a vacuum oven at 80 °C for 2 h. The process conditions involved a screw speed of 60 rpm and a temperature profile from the hopper to the die equal to 155–190–195–190–180 °C.

Moreover, 14 Basalt fabrics and PLA films were alternatively stacked (film stacking method) before being compression moulded at 180 °C with the following pressure cycle: 2 min at 0 bar, 2 min at 5 bar, 2 min at 15 bar, 2 min at 25 bar, and 2 min at 35 bar. After the moulding, a cooling step up to 30 °C was carried out with an applied pressure of 35 bar (the cooling cycle ends after 6 min). Composite samples were characterized by a symmetrical stacking sequence [0/90], an average thickness of 2.4 mm, and a target fibre content of approximately 45% by volume. The same procedure was used to manufacture glass-PLA composites that represented a benchmark. Composites were labelled as PLA-G (reinforced with glass fabrics), PLA-B (reinforced with neat basalt fabrics), and PLA-BZnO (reinforced with ZnO-decorated basalt fabrics).

### 2.7. Mechanical Characterization of Composite Laminates

Composites were characterized in tension (ASTM D3039) and in three-point bending (ASTM D790) with a Zwick/Roell Z010 (Zwick/Roell GmbH, Ulm, Germany). In tensile tests, a gauge length of 60 mm and a cross-head speed of 5 mm/min were used, while in bending tests, a span of 76 mm and a 5 mm/min cross-head speed were applied. Five tests were performed for each composite formulation.

### 2.8. Thermal Characterization

The decomposition temperature and the weight fraction of fibres in the composite materials were determined by thermogravimetric analysis (TGA) using a Setsys Evolution (Setaram, Caluire, France) instrument. Weighted amount of samples was inserted in alumina crucible and heated from room temperature (RT) to 800 °C at 10 °C min^−1^ in nitrogen atmosphere.

The thermal properties of neat PLA and PLA-fibre composites were studied by differential scanning calorimetry (DSC), with a Mettler Toledo DSC 822e (Mettler Toledo, Greisensee, Switzerland). The analysis was used to evaluate the transitions in heating and cooling scans, the initial crystallinity of the composites after their preparation and after annealing carried out at 80 °C for various time periods as well as the kinetics of isothermal melt crystallization. All the experiments were performed on about 10–15 mg of sample, weighted in aluminium pan, and under 30 mL min^−1^ N_2_ flux.

As regards the first set of experiments, the samples were heated from room temperature to 200 °C, cooled to RT and, then, reheated to 200 °C. All the temperature ramps were carried out at 10 °C min^−1^. Sample crystallinity *χ_c_* was determined in the first heating according to the Equation (1):

(1) $$\chi_{c}=\frac{\left(\Delta H_{m}-\Delta H_{cc}\right)}{W_{PLA}\;\Delta H_{m}^{0}}\times 100$$

where Δ*H_m_* and Δ*H_cc_* are the measured enthalpy of fusion and cold-crystallization, respectively, *W_PLA_* the polymer weight fraction obtained from TGA, and Δ*H_m_*^0^ = 93 J g^−1^ is the enthalpy of fusion of 100% crystalline PLA [47].

In the annealing experiments, the samples were heated at 30 °C min^−1^ from RT to 80 °C and kept at this temperature for predetermined times. Then, the samples were cooled at 30 °C min^−1^ to RT. Finally, in order to evaluate the conversion of the amorphous phase into the crystalline phase, a second scan was carried out at 10 °C min^−1^ up to 200 °C. The crystallinity evolution during the annealing was evaluated by the Equation (1) and the conversion of amorphous phase into crystalline phase according to the Equation (2):

(2) $$conversion\;=\frac{\left(\Delta H_{m}-\Delta H_{cc}\right)}{W_{PLA}\;\Delta H_{m}}\times 100$$

Isothermal melt crystallization experiments were carried out by heating the composite samples from RT to 200 °C and keeping them at this temperature for 3 min, in order to erase the previous thermal history. Then, the crystallization temperature, in the range of 105–122 °C, was reached at 30 °C min^−1^. The crystallization process was analysed by applying the Avrami equation (Equation (3)):

(3) $$X_{iso}=1-\exp\left(-kt^{n}\right)$$

where *n* is the exponent of time, depending upon the nucleation mechanism and growth morphology of the crystalline phase; *k* is the crystallization rate constant; and *X_iso_* is the relative crystallinity. It is defined as the ratio between the partial peak area at time *t* and the total exothermic peak area of the DSC trace, that is (Equation (4)):

(4) $$X_{iso}=\frac{\int_{0}^{t}\frac{dH}{dt}dt}{\int_{0}^{\infty }\frac{dH}{dt}dt}$$

where *dH/dt* is the heat flow recorded during crystallization. In preliminary experiments, used to check the effect on crystallization kinetics of any possible thermal degradation of the polymer in the melt state, the composites were melted also at 180 and 220 °C for 3 min before the isothermal crystallization at *T_c_* = 110 °C. Polarized optical microscopy (POM) analysis was performed by an Optiphot2-Pol light microscopy (Nikon) equipped with a Linkam HFS 91 hot stage (Linkam, Tadworth, UK) driven by a Linkam TP 92 temperature controller. The images were acquired by a Motic Image Plus 2.0 ML camera. Bare and ZnO-modified basalt fibres were immersed in a molten PLA film at 200 °C for 2 min. Then, the temperature was lowered at 120 °C at 50 °C min^−1^ and maintained at this temperature to follow the polymer isothermal melt crystallization.

## 3. Results and Discussion

### 3.1. Characterization of ZnO-Decorated Basalt Fabrics

**Figure 1:**
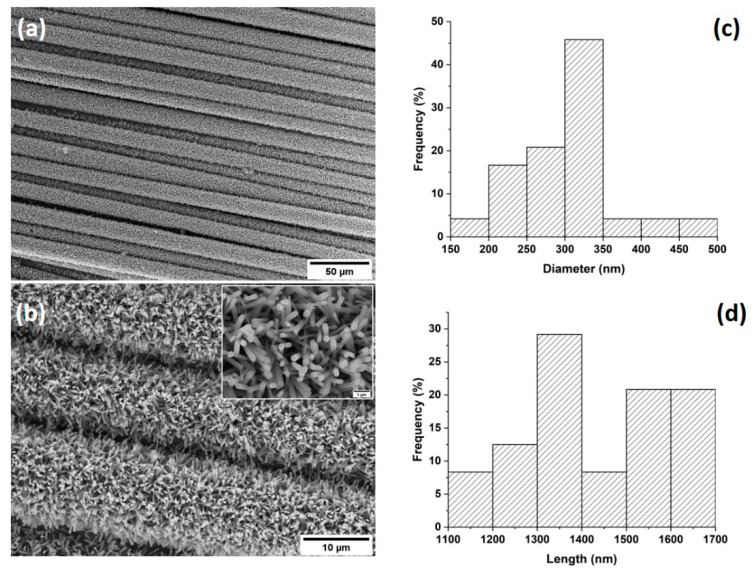
(**a**,**b**) SEM micrographs of basalt fabrics with ZnO nanorods at different magnifications and (**c**) diameter and (**d**) length distributions of ZnO nanorods.

Basalt fabrics were modified with ZnO through the hydrothermal process and were characterized morphologically by scanning electron microscopy. Figure 1a,b shows the SEM micrographs of the basalt fabrics after a 5 h growth process. From the micrographs, it is evident that ZnO hexagonal nanorods have grown homogeneously along the fibres, both axially and radially. Moreover, the growth of ZnO has been successfully and uniformly achieved on all the individual fibres of the fabric. From cross-sectional SEM micrographs (not included), it was possible to measure the diameters and lengths (Figure 1c,d) of the nanorods and the corresponding aspect ratio (L/d) that was approximately equal to 5, thus significantly increasing the surface area available for the interlocking mechanism with the polymer matrix.

**Figure 2:**
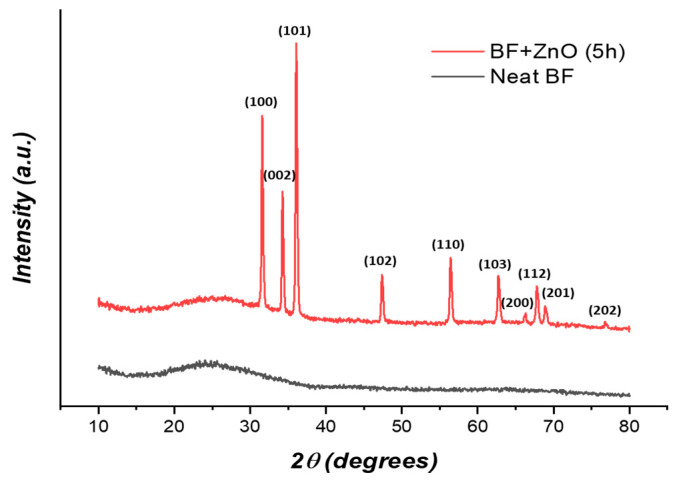
XRD spectra of the neat and ZnO-modified basalt fabrics.

X-ray diffraction was performed on neat and ZnO-modified basalt fabrics (Figure 2). XRD spectra showed that the neat basalt fabric is completely amorphous, while in the case of ZnO-modified fabrics, there is a clear presence of crystalline structures. The positions of the XRD peaks show good agreement with those of the hexagonal wurtzite structure of zincite [48].

**Figure 3:**
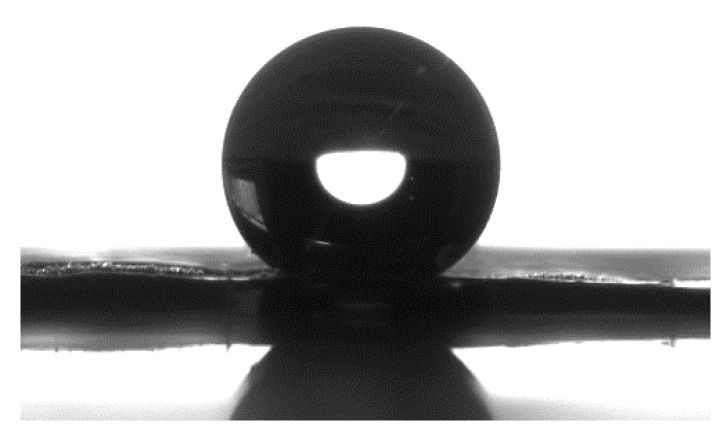
Water droplet test of basalt fabric modified with ZnO nanorods.

In addition to the increase in surface area of basalt fabrics, the presence of ZnO nanorods significantly changed the wettability of the material’s surface. Figure 3 shows that ZnO nanorods turned the hydrophilic behaviour of the neat fabric into a highly hydrophobic one, as evidenced by a contact angle value of 131°. The wettability of a surface is influenced by its morphology (surface roughness and micro-nanostructure) and the material-dependent surface energy, and in this regard, ZnO nanorods contributed to the development of hydrophobic properties by providing nanostructuring of the basalt fibres and increasing their roughness [27].

### 3.2. Thermal Characterization of PLA-Based Composites

**Figure 4:**
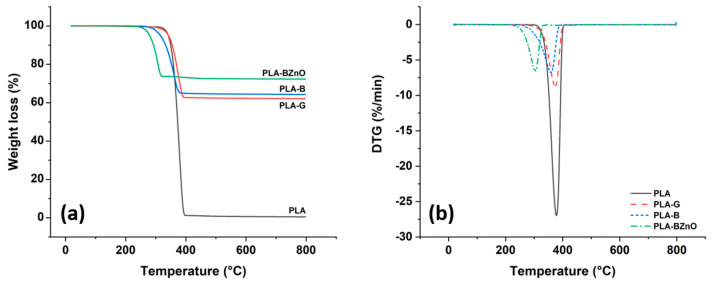
(**a**) TGA and (**b**) DTG thermograms of neat polymer and composites.

The amount of fibres in each composite formulation and their thermal stability were determined by TGA. Selected thermograms of the neat PLA and of three laminates are reported in Figure 4, while the results of the TGA experiments, including the PLA content, the temperature at 5% and 10% decomposition (*T_d_^5%^* and *T_d_^10%^*), and the temperature at maximum decomposition rate (*T_d_^max^,* from derivative thermogravimetry (DTG)) are summarized in Table 1.

The thermogram of neat PLA shows that it completely decomposes between about 300 and 400 °C, in good agreement with the results reported in literature [49]. A similar temperature decomposition range can be observed for the PLA-B and PLA-G samples, even though some differences can be pointed out depending on the fibre type.

The value of the temperature for 5% weight loss, which is generally considered to be the initial decomposition temperature, was shifted to lower temperature when basalt fibres were incorporated in a PLA matrix, and this can be extended to the other relevant temperatures indicated in Table 1. On the contrary, glass fibres did not modify the thermal stability of neat PLA. It is known that PLA is very sensitive to temperature and hydrolysis during melt-processing [50]. In particular, the degradation of PLA is often ascribed to hydrolysis by trace amounts of water and random main-chain scission during melt-processing. Recently, Mazzanti et al. [51] showed how even low amounts of natural fibres can degrade the PLA matrix due to the presence of a limited moisture content during melt-processing. It is likely that the presence of some residual moisture due the polar character of basalt fibres induced hydrolysis of the ester bonds along the backbone of the PLA chain. This thermal degradation is even more exacerbated by the presence of ZnO nanorods. In this case, zinc compounds are known to catalyse both the intermolecular transesterification reactions, leading to PLA with lower molecular weights, and the “unzipping” depolymerization [42,52] with the selective formation of lactide. On the contrary, when Zn-based compounds are absent, PLA degradation can be considered the result of a competition between the random chain scission via a cis-elimination reaction and the cyclic rupture via intramolecular transesterification of the molecules. Despite these degradative effects, the onset of thermal decomposition is still higher than 270 °C.

**Figure 5:**
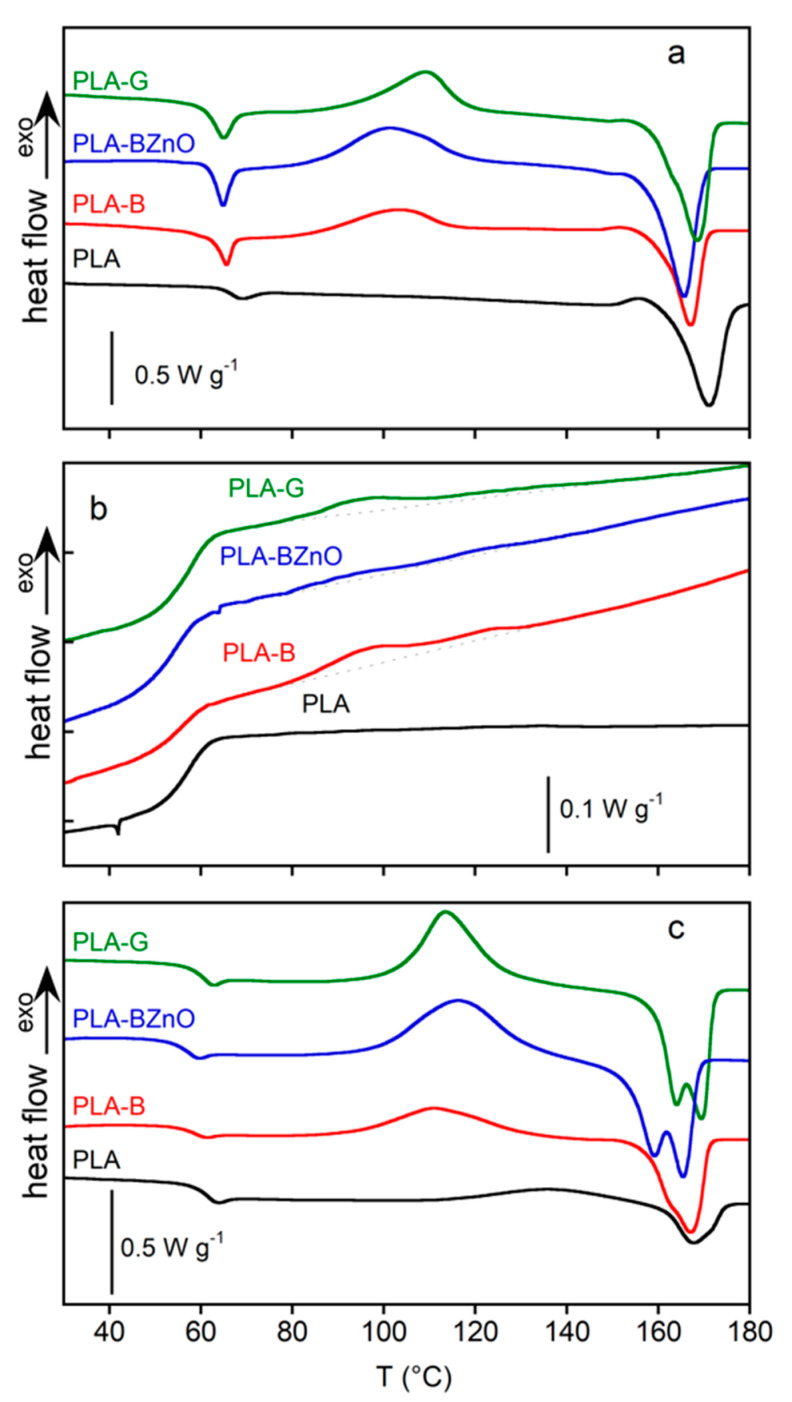
Differential scanning calorimetry (DSC) profiles of neat poly(lactic acid) (PLA) and composites recorded at 10 °C min^−1^. First heating (**a**), first cooling (**b**), and second heating (**c**).

The DSC thermograms of three selected composite samples and as received PLA, acquired in the three temperature ramps (first heating, cooling and second heating), are displayed in Figure 5. In Table 2, the glass transition temperature onset (*T_g_^os^*), the onset (*T_cc_^os^*), and peak temperature of the cold crystallization (*T_cc_*) and melting processes (*T_m_*) as well as the enthalpies involved in the transitions Δ*H_cc_* and Δ*H_m_*, all recorded in the first heating scan, are reported. As regards PLA, the results of the second heating scan should be used for comparison with the thermal properties of composites, because the neat PLA samples did not experience the same thermal history of the laminates. In the low temperature range, all the composite samples show the glass transition superimposed to an overshoot peak, due to the physical aging occurred at room temperature. The *T_g_^os^* of the composites, located at about 56 °C, is not affected by the nature of the fibres, thereby suggesting that the presence of fibres had a limited effect on the chain mobility of PLA. After the glass transition, the cold crystallization of composites occurred at temperature lower than that showed by the neat PLA sample in the second heating, which is characterized by a weak and broad cold crystallization peak, centred at about 136 °C (Figure 5c). The low *T_cc_^os^* and *T_cc_* of composites indicate that the nucleation stage of the crystallization process is favoured directly by the fibre surface or, indirectly, by the crystalline layer possibly grown on the fibre surface during the cooling stage of laminate preparation. At higher temperature, the polymer melts at about 164–169 °C. The data about cold crystallization and melting enthalpies, obtained by sampling the composites in different zones, resulted quite scattered because of the inherent nonhomogeneous nature of composites.

**Figure 6:**
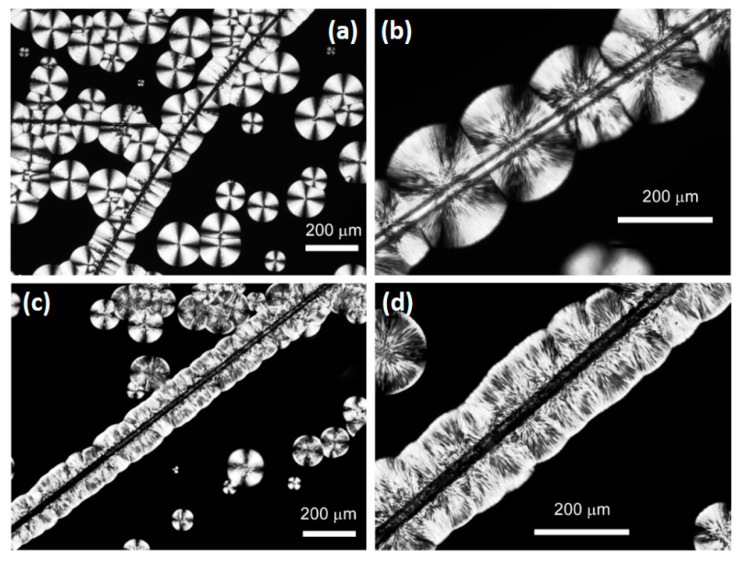
Polarized optical microscopy (POM) images of poly(lactic acid)-reinforced with neat basalt fibres (PLA-B) (**a**,**b**) and poly(lactic acid)-reinforced with ZnO-decorated basalt fibres (PLA-BZnO) (**c**,**d**) crystallized at 120 °C for 20 min and acquired at magnification 40× (**a**,**c**) and 100× (**b**,**d**).

In the cooling scan, the neat PLA did not crystallize while the composites DSC profiles show a very weak and broad exothermic process characterized by two peaks at about 96 and 122 °C (in Figure 5b, the baselines were reported to highlight the transitions). In general, a very low crystalline fraction was obtained by cooling the samples at 10 °C min^−1^. The PLA-BZnO sample reached a lower crystallinity (*χ_c_* ~ 1%) with respect to PLA-B (*χ_c_* ~ 4%) and PLA-G (*χ_c_* ~ 3%) composites. In the second heating, the cold crystallization of composites began at temperature always lower than that of the PLA, confirming the nucleating effect of the fibre surface. The maximum crystallinity reached by the neat PLA and composites in the second heating, in which all the samples were subjected to the same controlled thermal history, can be evaluated by the ratio between the measured melting enthalpy and Δ*H_m_^0^*. The obtained values, 12%, 30%, 52%, and 48% for PLA, PLA-B, PLA-BZnO, and PLA-G, respectively, clearly show that the presence of the fibres favoured the crystallization process. Actually, the nucleating activity of glass and basalt fibres on PLA matrix is well documented in literature [10,45], but no indication is reported on ZnO nanorods sizing. In order to observe directly the effect of the surface modification, polarized optical microscopy (POM) analysis was carried out. In particular, the PLA isothermal melt-crystallization process at 120 °C was followed in presence of bare and ZnO-modified basalt fibres. In Figure 6, the POM images, recorded after 20 min, are reported.

All the POM images show clear transcrystallization phenomena due to the nucleating activity of both fibre surfaces. However, a different morphology, related to the different nucleation densities, can be observed. In the PLA-B sample, a sporadic nucleation occurs on bare basalt fibre and the spherulite structure can be observed. This result is very similar to that described by Pan et al. [46] related to the same PLLA4032D crystallization in presence of basalt fibres. On the other hand, the nucleation density on ZnO surface is so high that the spherulite cannot be distinguished but a columnar morphology appeared. ZnO nanoparticles as nucleating agents were widely reported in literature but there is not a systematic study on their efficiency [53,54,55,56,57]. In general, compared to other inorganic nucleating agents or silanized ZnO, ZnO nanoparticles dispersed in PLLA matrix show a moderate nucleation activity. However, the high specific surface area of the ZnO nanorods on basalt surface brings about the observed high nucleation density.

**Figure 7:**
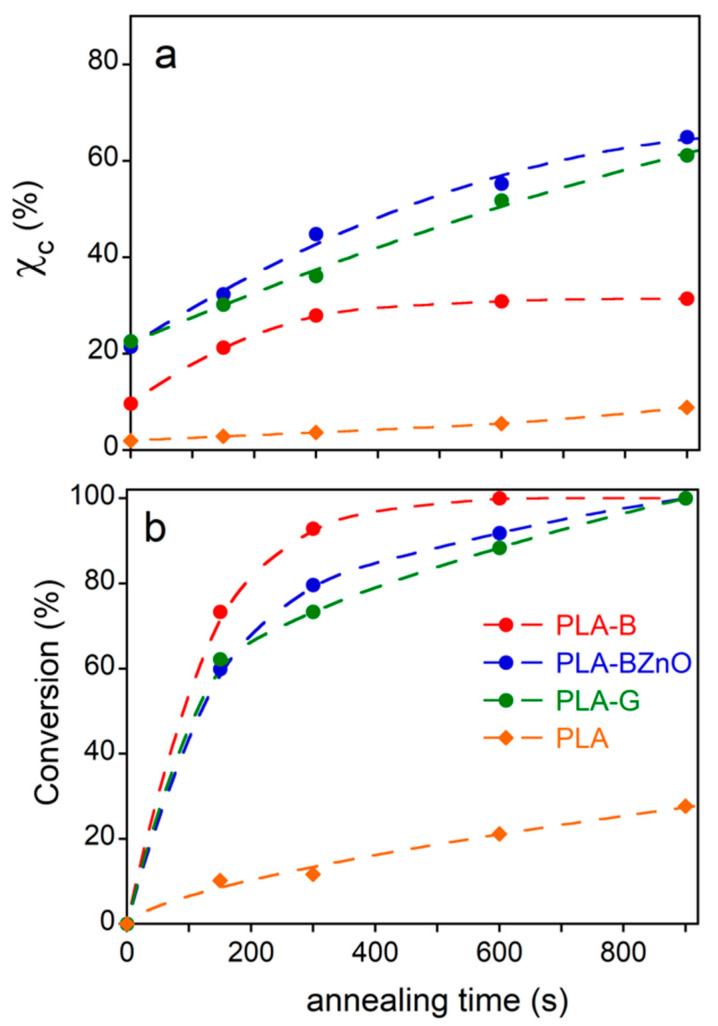
(**a**) Crystallinity (*χ_c_*) and **(b**) conversion of neat polymer and composites as a function of annealing time at 80 °C. The curves are guide for the eye.

As shown in Figure 5, the crystallinity of the laminate matrix is low, although, in comparison to the neat polymer, the presence of the fibres decreases the cold crystallization temperature and favours a partial crystallization process during the cooling stage of material’s manufacturing. In order to follow the composite further crystallization process, the samples were subjected to an annealing at 80 °C for different times. In these experiments, it was not possible to directly elaborate the kinetics of cold crystallization from DSC data. In fact, most part of initial exothermal heat flow at the onset of crystallization was superimposed to the instrumental lag time necessary to reach the temperature equilibrium, being very short the crystallization induction period of the already nucleated semicrystalline polymer. Therefore, the evolution of crystallinity and the conversion at each annealing time was determined from the cold and melting enthalpy, evaluated in a subsequent heating run, according to the Equations (1) and (2), respectively, and reported as a function of the annealing time in Figure 7a,b. After 900 s, the annealing at 80 °C of the PLA-B, PLA-BZnO, and PLA-G samples resulted in a complete conversion, being absent the exothermic process of the cold crystallization in the last heating scan. Differently, the conversion and the crystallinity of the neat polymer were low in all the explored time range. In agreement with the data reported in Table 2 and with the melting enthalpy acquired in the DSC second heating, it can be observed that the crystallinity of samples with basalt fibres was always lower than that obtained by the other two composites, from the initial value up to the complete conversion, occurring at 600 s. The PLA-BZnO and PLA-G reached the highest crystallinity (*χ_c_* ~ 60%) at the end of the thermal treatment.

**Figure 8:**
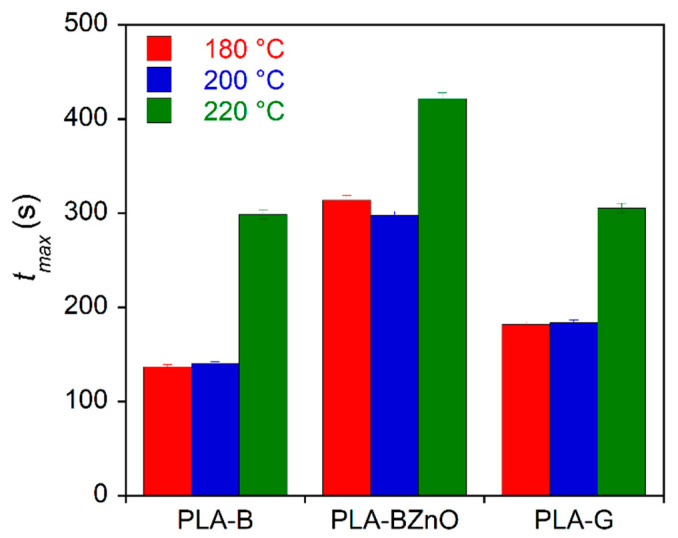
Time where the crystallization rate is maximum at *T*_c_ = 110 °C after melting the composite samples at 180, 200, and 220 °C for 3 min.

In addition, the influence of fabrics on the PLA crystallization kinetics was evaluated by isothermal melt crystallization experiments carried out between *T_c_* = 105 °C and *T_c_* = 122 °C. It is well-known that, as other polyesters, PLA can undergo degradation and chain scission at a few degrees above the melting point. Moreover, the thermogravimetric experiments (Figure 4) showed that the presence of ZnO nanorods catalyses the polymer decomposition. Then, in order to evaluate any possible effect of a further thermal treatment on crystallization kinetics, the composites were melted for 3 min at 180, 200, and 220 °C before the isothermal step, carried out at the selected *T_c_* = 110 °C. In Figure 8, the time at which the crystallization rate is maximum (*t_max_*), that is in correspondence of the exothermic peak, is reported for the three composite samples and the three explored temperatures.

**Figure 9:**
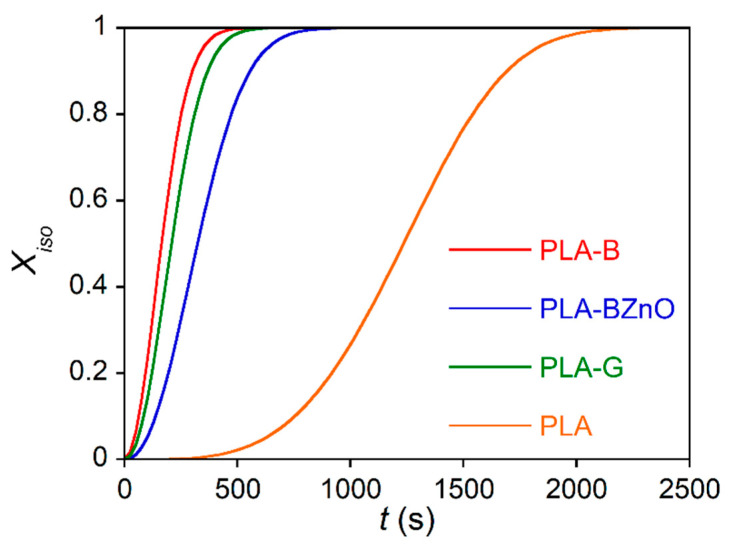
Relative crystallinity evaluated in the isothermal crystallization at *T*_c_ = 110 °C of neat PLA and composite samples.

The plot clearly shows that the most severe thermal treatment at 220 °C resulted in a drastic crystallization rate slowdown of all the samples, while at 180 and 200 °C, the melting temperature did not affect the process kinetics. Then, in the following experiments, the isothermal crystallization was carried out using a residence temperature of 200 °C of the polymer in the melt state. From the DSC thermograms, the conversion of the amorphous phase into the crystalline one was evaluated as a function of the crystallization time according to the Equation (4). As an example, in Figure 9, the relative crystallinity curves of the laminates and neat polymer obtained at *T_c_* = 110 °C are reported.

The data of *X_iso_* vs. *t* were interpolated by the Avrami equation (Equation (3)) to obtain the kinetics constant *k* and the exponent *n*. The data, shown in Table 3, are reported as the mean value ± standard deviation on at least 3 repetitions.

The mean Avrami coefficient *n* = 3.6 found in PLA experiments suggests that the crystalline phase of neat polymer grew by forming spherulites from a heterogeneous and homogeneous mixed nucleation, because of the possible presence of impurities that act as heterogeneous nuclei. By comparing the values reported in Table 3, the first observation concerns the decrease in *n* value of the composites (*n* = 2.0–2.1) with respect to the neat polymer, indication of a change in nucleation and/or crystalline growth mechanism.

**Figure 10:**
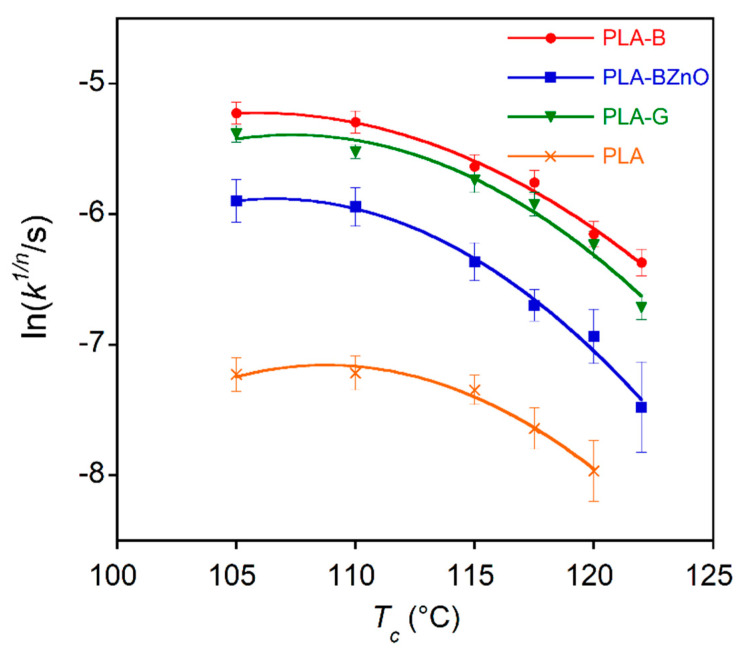
Variation of the isothermal melt crystallization rate (ln(*k^1/n^*)) of neat polymer and composites as a function of crystallization temperature. The lines are guide for the eye.

In fact, the POM experiments evidenced a clear transcrystalline zone around the fibres, due to a high nucleation density of the filler surface. According to the computer simulation model proposed by Krause et al. [58], in this condition, a *n* value between 2 and 2.6 is expected. Different results have been obtained by Liu et al. [59], who reported mean *n* values of 2.8 and 2.9 for neat PLA and basalt fibre-loaded PLA, respectively. However, the differences could be due to the lower fibre amount (2 wt.%) used in that research. In order to compare the crystallization kinetics of the composites and neat PLA, in Figure 10, the ln(*k^1/n^*) as a function of the crystallization temperature is reported. The figure shows that the composites crystallized faster than the neat polymer, mainly those containing glass and basalt fabrics, while the PLA-BZnO sample showed a kinetics slower than the other two composites. However, the neat polymer and composites, at the end of the crystallization process, reached nearly the same final crystallinity (*χ_c_* = 36 ± 3%), evaluated from the integrated enthalpy of the isothermal crystallization process.

A possible polymer partial degradation occurring during laminate preparation, favoured by the presence of ZnO nanorods, could explain the slower crystallization rate of the PLA-BZnO composite. In addition, the micro-/nanostructure of the ZnO rods can obstacle the diffusion of the PLA chains through a pinning effect and then, slow down the growth of crystalline phase. Similar retardation effects on polymer crystallization have been reported by Yu et al. [60], Diez-Pascual [61], and Diez-Vicente [62] in poly(3-hydroxybutyrate-co-3-hydroxyvalerate)/ZnO nanocomposites. The authors suggested that it is due to the strong interactions between the hydroxyl groups of the ZnO surface and the C=O of the ester groups of polymer that hamper the macromolecule mobility and, then, the crystal growth. The slow-down effect of the ZnO nanorods did not act in the cold crystallization experiments carried out at 80 °C (Figure 7), being the chain mobility mainly reduced by the polymer viscosity increase at low temperature.

Definitively, the presence of the ZnO on fibre surface influences the polymer melt-crystallization by two opposite effects. In fact, it favours the nucleation activity with respect to bare basalt fibres but, on the other hand, slows down the overall crystallization rate, presumably because of the possible partial degradation or reduced mobility of PLA chains close to the ZnO nanorods. Differently, in the cold-crystallization process, the formation of a pre-existing crystalline phase, presumably grown around the modified fibre during the composite manufacturing, speeds up the process.

### 3.3. Mechanical Characterization of Composites

**Figure 11:**
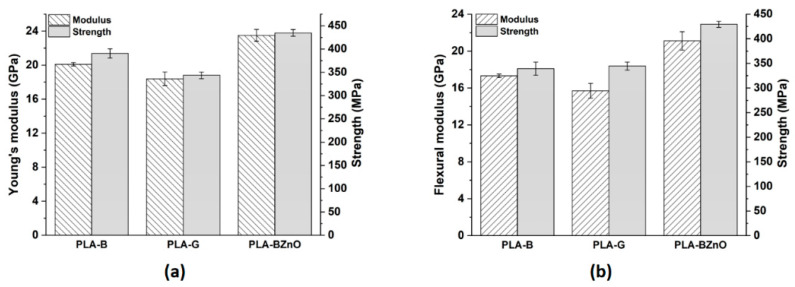
(**a**) Tensile and (**b**) flexural properties of PLA-based composites.

The tensile properties of the composites under investigation are summarized in Figure 11. In both loading conditions, basalt fibres show a better reinforcing effect compared to glass, while the presence of ZnO nanorods resulted to be beneficial not only to the stiffness but also to the strength.

**Figure 12:**
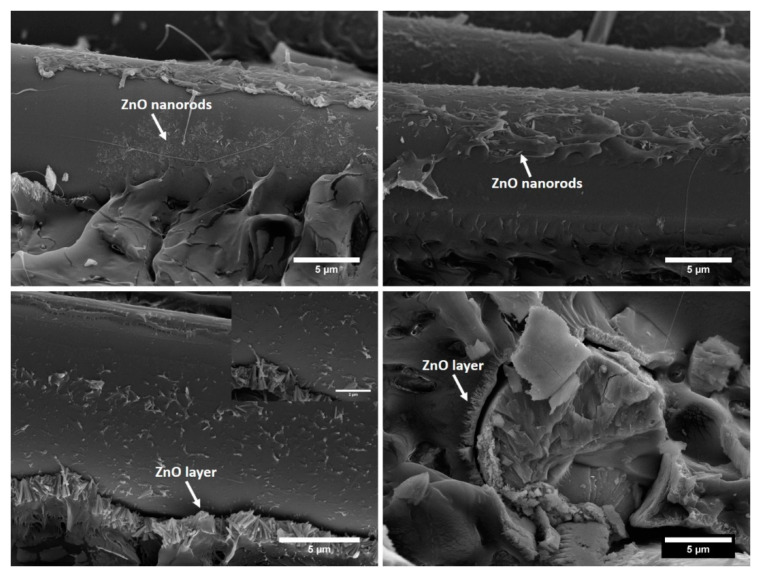
SEM micrographs detailing the fracture surface of PLA-BZnO composites.

In tension, the stiff ZnO nanorods increased the stiffness of 17% and 28% compared to neat basalt and glass fabrics, respectively, while the strength improvement was around 11% and 26% with respect to PLA-B and PLA-G composites, respectively. In bending, ZnO nanorods resulted in even higher increases in stiffness (22% and 34%) and strength (26% and 24%) compared to neat basalt and glass fibre reinforced composites, respectively. The modulus improvement with the ZnO nanofiller is in line with the results available in literature. In [42], a 3 wt% of ZnO in PLA matrix was enough to grant a 24% increase in Young’s modulus, but this was associated with a slight decrease in tensile strength. Recently, He et al. [20] designed SiO_2_/PCL hybrid coatings on basalt fibres in order to increase simultaneously the Young’s modulus and tensile strength of basalt fabric-reinforced PLA composites. In optimized formulations, a tensile modulus of 9 GPa and a tensile strength of 421 MPa have been achieved for a basalt fibre content by volume of 49.5%. It is clear that the incorporation of ZnO nanorods on the surface of basalt fibres was particularly effective in improving stiffness and strength. As detailed in Section 3.2, both basalt and glass fabrics showed a nucleating activity for PLA, therefore, the increase in mechanical properties can be also ascribed to the enhanced crystallinity of the polymer matrix. It is interesting to note that the degradation of PLA promoted by ZnO nanorods did not result in the deterioration of the composites mechanical properties. This negative effect was more than balanced by the better interfacial adhesion, as can be seen in the SEM micrographs of the fracture surface (Figure 12).

It was observed that ZnO nanorods were successfully wetted by PLA and embedded in the PLA matrix. Basalt fibres show extensive presence of PLA matrix adhered to the surface, and many instances of interfacial cohesive failure were detected, with the simultaneous occurrence of ZnO and traces of PLA matrix. Stiff ZnO nanorods adhered to the basalt fibres surface and increased the surface roughness, thereby promoting the mechanical interlocking between fibres and matrix. This resulted in an improved interfacial strength that delayed the interfacial debonding. This effect was likely enhanced by the compact transcrystallinity structure typical of hierarchical basalt fibres (Figure 6d) that can favour the stress transfer from the matrix to the fibre [46]. Under tensile loading, especially for fibres directly loaded in tension, basalt fibre failure in the fracture plane was observed where, with increasing deformation, the high stress transferred to the rigid ZnO nanorods and the corresponding stress concentration could not be relieved and detachment of ZnO layer from the fibres occurred.

**Figure 13:**
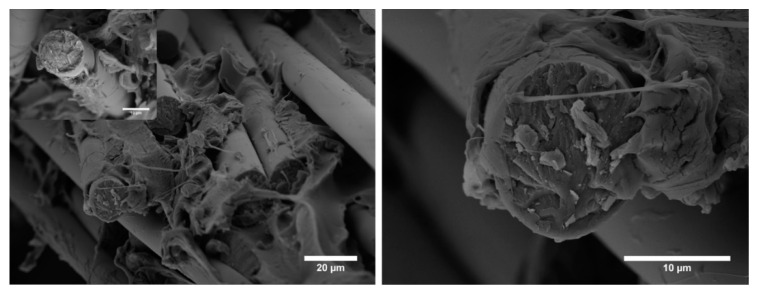
SEM micrographs detailing the fracture surface of PLA-B composites.

On the contrary, neat basalt fibre composites featured a lower interfacial adhesion, with evidence of extensive fibre pull-out and lower adherence of PLA matrix on the fibre surface, despite being present due to the silane coupling agent (Figure 13).

**Figure 14:**
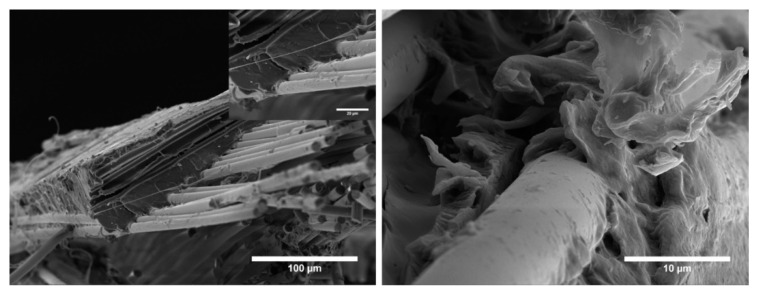
SEM micrographs detailing the fracture surface of poly(lactic acid)-reinforced with glass fabrics (PLA-G) composites.

Complete debonding at the fibre/matrix interface is absent also in glass fibre reinforced PLA composites (Figure 14), but pull-out phenomena dominated the fracture surface, thus confirming the lower mechanical properties achieved by this composite formulation.

## 4. Conclusions

This work demonstrates that it is possible to obtain competitive PLA-based laminates for at least semistructural applications using commercially available basalt-woven fabrics and PLA, especially when the PLA/basalt fibre interface is accurately tailored. In this context, the surface decoration of basalt fibres with ZnO nanorods by a low-temperature hydrothermal process was successfully developed and used to modify the interfacial adhesion with a PLA matrix. The presence of ZnO with a hexagonal wurtzite structure resulted in basalt fabrics with a hydrophobic behaviour and in PLA degradation, but, at the same time, promoted the formation of a compact transcrystallinity structure that, coupled with the mechanical interlocking action, enabled an efficient stress transfer at the interface. Composites with strength and stiffness in the order of 430 MPa and 23 GPa, respectively, were obtained, thus realizing the potential of high-performance basalt fibre/PLA composites. From crystallization studies, it was found that ZnO affects the polymer melt-crystallization by promoting the nucleation activity compared to neat basalt fibres but, on the other hand, retards the overall crystallization rate, likely due to the partial degradation or reduced mobility of PLA chains close to the ZnO nanorods.

## Figures and Tables

**Table 1:** Temperatures at 5% (*T_d_^5%^*) and 10% decomposition (*T_d_^10%^*), temperature at maximum decomposition rate (*T_d_^max^*) and poly(lactic acid) (PLA) content in composites (*W_PLA_*) obtained from TGA experiments. The data are reported as mean values ± SD (*n* = 3).

Sample	*T*_d_^5%^ (°C)	*T*_d_^10%^ (°C)	*T*_d_^max^ (°C)	W_PLA_ (wt.%)
PLA	341.4 ± 0.5	355.2 ± 0.4	372.1 ± 0.8	100
PLA-B	314.6 ± 1.2	332.1 ± 1.0	357.8 ± 1.4	34.3 ± 0.6
PLA-BZnO	278.7 ± 1.1	291.4 ± 1.3	302.2 ± 1.8	25.4 ± 1.3
PLA-G	342.5 ± 0.2	354.4 ± 0.3	372.9 ± 1.2	36.6 ± 0.4

**Table 2:** Thermal properties of PLA and PLA-based composites determined from differential scanning calorimetry (DSC) experiments. The values are reported as mean values ± SD on at least 5 samples.

Sample	*T*_g_^os^ (°C)	*T*_cc_^os^ (°C)	*T*_cc_ (°C)	*T*_m_ (°C)	Δ*H*_cc_ (J g^−1^)	Δ*H*_m_ (J g^−1^)	*χ*_c_ (%)
PLA second heating	57 ± 1	109 ± 1	134 ± 2	166 ± 1	6.5 ± 0.2	9.3 ± 0.4	3 ± 1
PLA-B first heating	57 ± 3	77 ± 3	101 ± 3	164 ± 2	26 ± 5	37 ± 3	12 ± 8
PLA-BZnO first heating	56 ± 3	76 ± 2	101 ± 3	165 ± 1	30 ± 6	50 ± 5	21 ± 11
PLA-G first heating	56 ± 3	81 ± 1	108 ± 1	168 ± 1	21 ± 5	42 ± 6	22 ± 13

**Table 3:** Kinetic constants *k* and *n* of neat polymer and composites evaluated in isothermal experiments carried out at different temperatures *T_c_*.

Crystallization Temperature	PLA-B		PLA-BZnO		PLA-G		PLA	
*T*_c_ (°C)	k (s^−^^n^)	*n*	k (s^−^^n^)	n	k (s^−^^n^)	*n*	k (s^−^^n^)	*n*
105	(2.1 ± 0.01) 10^−5^	2.16 ± 0.02	(2.18 ± 0.05) 10^−5^	1.82 ± 0.02	(2.76 ± 0.1) 10^−5^	1.95 ± 0.03	(2.1 ± 0.05) 10^−11^	3.4 ± 0.6
110	(2.7 ± 0.08) 10^−5^	2.05 ± 0.01	(1.86 ± 0.05) 10^−6^	2.2 ± 0.1	(8.71 ± 0.03) 10^−6^	2.10 ± 0.02	(1.0 ± 0.06) 10^−13^	3.8 ± 0.1
115	(3 ± 0.1) 10^−6^	2.23 ± 0.02	(3.16 ± 0.06) 10^−6^	2.0 ± 0.1	(1.0 ± 0.5) 10^−5^	2.04 ± 0.05	(1.3 ± 0.04) 10^−11^	3.4 ± 0.2
117.5	(7 ± 0.01) 10^−6^	2.02 ± 0.01	(2.28 ± 0.02) 10^−6^	1.92 ± 0.01	(9.6 ± 0.1) 10^−6^	1.95 ± 0.03	(1.9 ± 0.3) 10^−11^	3.2 ± 0.2
120	(6 ± 0.02) 10^−6^	1.95 ± 0.02	(1.89 ± 0.04) 10^−6^	1.96 ± 0.02	(1.2 ± 0.1) 10^−6^	2.19 ± 0.05	(1.4 ± 0.5) 10^−14^	4.0 ± 0.1
122	(8 ± 0.2) 10^−7^	2.21 ± 0.01	(7.2 ± 0.1) 10^−8^	2.2 ± 0.1	(9 ± 1) 10^−8^	2.41 ± 0.05	–	–
Mean		2.1 ± 0.1		2.0 ± 0.2		2.1 ± 0.2		3.6 ± 0.3

## Data Availability

The data presented in this study are available on request from the corresponding author.
